# Ductal Dilatation of ≥5 mm in Intraductal Papillary Mucinous Neoplasm Should Trigger the Consideration for Pancreatectomy: A Meta-Analysis and Systematic Review of Resected Cases

**DOI:** 10.3390/cancers13092031

**Published:** 2021-04-22

**Authors:** Y.H. Andrew Wu, Atsushi Oba, Laurel Beaty, Kathryn L. Colborn, Salvador Rodriguez Franco, Ben Harnke, Cheryl Meguid, Daniel Negrini, Roberto Valente, Steven Ahrendt, Richard D. Schulick, Marco Del Chiaro

**Affiliations:** 1 Division of Surgical Oncology, Department of Surgery, University of Colorado Anschutz Medical Campus, Aurora, CO 80045, USA; YUAN-HAW.WU@CUANSCHUTZ.EDU (Y.H.A.W.); ATSUSHI.OBA@CUANSCHUTZ.EDU (A.O.); LAUREL.BEATY@CUANSCHUTZ.EDU (L.B.); KATHRYN.COLBORN@CUANSCHUTZ.EDU (K.L.C.); SALVADOR.RODRIGUEZFRANCO@CUANSCHUTZ.EDU (S.R.F.); CHERYL.MEGUID@CUANSCHUTZ.EDU (C.M.); dan_negrini2000@yahoo.com.br (D.N.); robbie.valente@gmail.com (R.V.); STEVEN.AHRENDT@CUANSCHUTZ.EDU (S.A.); RICHARD.SCHULICK@CUANSCHUTZ.EDU (R.D.S.); 2 Department of Hepatobiliary and Pancreatic Surgery, Cancer Institute Hospital, Japanese Foundation for Cancer Research, Tokyo 135-8550, Japan; 3 Department of Biostatistics and Informatics, University of Colorado Anschutz Medical Campus, Aurora, CO 80045, USA; 4 Surgical Outcomes and Applied Research Program, University of Colorado Anschutz Medical Campus, Aurora, CO 80045, USA; 5 The Heart Institute, Children’s Hospital Colorado, Aurora, CO 80045, USA; 6 Strauss Health Sciences Library, University of Colorado Anschutz Medical Campus, Aurora, CO 80045, USA; BEN.HARNKE@CUANSCHUTZ.EDU; 7 Department of Anesthesiology, Federal University of the State of Rio de Janeiro, Rio de Janeiro 21941-901, Brazil; 8 Department of Surgery and Perioperative Sciences, Umeå University Hospital, 907 37 Umeå, Sweden; 9 University of Colorado Cancer Center, Aurora, CO 80045, USA

**Keywords:** pancreatic main duct dilatation, intraductal papillary mucinous neoplasm, high grade dysplasia, invasive carcinoma, pancreatic cystic neoplasm, pancreatic cancer, meta-analysis

## Abstract

**Simple Summary:**

Intraductal papillary mucinous neoplasms (IPMN) are common but difficult to manage since accurate tools for diagnosing malignancy are unavailable. This study evaluates the diagnostic value of main pancreatic duct (MPD) diameter for detecting IPMN malignancy, using a meta-analysis of published data. The result suggests that malignancy is highly prevalent in IPMN with ductal dilatation of >5 mm.

**Abstract:**

Intraductal papillary mucinous neoplasms (IPMN) are common but difficult to manage since accurate tools for diagnosing malignancy are unavailable. This study tests the diagnostic value of the main pancreatic duct (MPD) diameter for detecting IPMN malignancy using a meta-analysis of published data of resected IPMNs. Collected from a comprehensive literature search, the articles included in this analysis must report malignancy cases (high-grade dysplasia (HGD) and invasive carcinoma (IC)) and MPD diameter so that two MPD cut-offs could be created. The sensitivity, specificity, and odds ratios of the two cutoffs for predicting malignancy were calculated. A review of 1493 articles yielded 20 retrospective studies with 3982 resected cases. A cutoff of ≥5 mm is more sensitive than the ≥10 mm cutoff and has pooled sensitivity of 72.20% and 75.60% for classification of HGD and IC, respectively. Both MPD cutoffs of ≥5 mm and ≥10 mm were associated with malignancy (OR = 4.36 (95% CI: 2.82, 6.75) vs. OR = 3.18 (95% CI: 2.25, 4.49), respectively). The odds of HGD and IC for patients with MPD ≥5 mm were 5.66 (95% CI: 3.02, 10.62) and 7.40 (95% CI: 4.95, 11.06), respectively. OR of HGD and IC for MPD ≥10 mm cutoff were 4.36 (95% CI: 3.20, 5.93) and 4.75 (95% CI: 2.39, 9.45), respectively. IPMN with MPD of >5 mm could very likely be malignant. In selected IPMN patients, pancreatectomy should be considered when MPD is >5 mm.

## 1. Introduction

Pancreatic cystic lesions are common. The prevalence of these lesions is around 50% in the general population and increases with age [1]. Of these cystic lesions, pancreatic cystic neoplasm (PCN) management is challenging and important in modern pancreatology. The challenge lies in the difficulty in accurately discerning completely benign PCNs from ones with potential for malignancy progression [2,3,4,5].

Intraductal papillary mucinous neoplasm (IPMN) is a pre-cancerous lesion that accounts for at least half of all PCNs [6]. Morphologically, IPMNs can be divided into two major categories: (1) the branch-duct IPMN (BD-IPMN) that only involves the peripheral pancreatic ducts, and (2) main-duct IPMN (MD-IPMN) and mixed-type IPMN that involve the main pancreatic duct (MPD) and/or the branch ducts [7]. The morphologic and radiologic classification for identifying BD-IPMN, MD-IPMN, or mixed-type IPMN is extremely important. It is well known that BD-IPMNs have a very low risk for cancer progression; in contrast, MD- and mixed-type IPMNs are more prone to becoming cancerous [2,8]. Studies have shown that it is safe to surveil small BD-IPMNs (below 3–4 cm) that do not have any radiological features suggestive of malignancy [8,9,10]. In contrast, IPMNs involving the MPDs are more aggressive and generally need to be treated surgically [6,11].

The International Consensus Guidelines and the European Guidelines for managing IPMNs concordantly suggest an aggressive approach for managing MD- and mixed-type IPMNs. However, the role of MPD dilatation in deciding the indication for surgical resection of IPMN is moderately different in the two guidelines. The International Consensus Guidelines for IPMN management suggests that MD- or mixed-type IPMN with MPD dilatation of 10 mm or more should be referred for surgical treatment (high-risk stigmata) [11]. However, the same guideline suggests that surgery could still be considered for patients with 5–9.9 mm MPD (a worrisome feature), if there is the presence of mural nodule(s) ≥5 mm, cytology positivity for malignancy, or main-duct features suspicious for malignancy involvement [11]. The first European Guideline, published in 2013, recommended lowering the cutoff of MPD dilatation for surgery indication from 10 mm to 6 mm [2]. This approach was later supported by retrospective analysis that showed the implementation of MPD dilatation of 10 mm as a cutoff for surgery indication risks the possibility of IPMN undertreatment, as patients could have already developed invasive carcinoma (IC) or high-grade dysplasia (HGD) [12,13,14]. For better management of IPMNs, the latest evidence-based European Guidelines, published in 2018, recommends surgical resection of IPMN with MPD dilatation of 5–9.9 mm, if the patient is fit and has a long life expectancy (relative indications for surgery) [6]. This approach has also been recently supported by large retrospective surgical analyses [15].

Meanwhile, some studies have demonstrated a safe conservative approach in managing patients with suspected MD- or mixed-type IPMNs that have MPD dilatation of 5–9.9 mm [16,17]. Despite the compelling data, those studies need to be reassessed since high-volume centers have demonstrated that the accuracy of the pre-operative diagnosis of PCNs is approximately 60–80% [18,19,20]. Therefore, a high percentage of the suspected MD- or mixed-type IPMNs in those studies could very likely represent other more benign pancreatic diseases, such as chronic pancreatitis [18,19,20]. Hence, the optimal study design to analyze the disease status of PCNs is to evaluate a cohort with available histology data [21].

The role of MPD dilatation is important for establishing surgical indication. However, currently there is no consensus across existing guidelines regarding the degree of ductal dilatation that warrants pancreatectomy. This study is designed to evaluate the association of HGD and IC with ≥5 mm and ≥10 mm MPD. Sensitivity and specificity for detecting HGD and IC were calculated using the two MPD cutoffs.

## 2. Materials and Methods

### 2.1. Article Search/Selection and Outcome Assessment

This systematic review and meta-analysis was performed by following the Preferred Reporting Items for Systematic Reviews and Meta-Analyses (PRISMA) Guideline [22]. Literature search was performed on 28 February 2020 by a professional librarian at the Strauss Health Sciences Library. The following databases were queried: Ovid MEDLINE(R) ALL 1946 to 27 February 2020; Embase; Web of Science; Google Scholar. The search strategy focused on obtaining all existing literature related to MPD dilatation in IPMN and its association to malignancy. The terms including but not limited to: intraductal papillary mucinous or intraductal mucinous papillary or ipmn or ipmt; dilat or size or diameter or cut off or cutoff or mm or millimeter or milli meter were used for the systematic search. Full search strategy is provided in online Figure S1. The articles obtained from the search were uploaded to the Endnote version X9 citation management application, in which duplicated search results were eliminated. The final list of articles for initial review was uploaded to Covidence, a software designed for reviewing and selecting articles for systematic review and meta-analysis.

In the initial phase of reviewing articles, Y.H.A.W. and A.O. independently studied all records uploaded to Covidence. If the title or abstract of the articles were relevant to the topic of this study, the independent reviewers (Y.H.A.W. and A.O.) would thoroughly perform a screening of the entire article to assess for eligibility of inclusion. Non-English articles were excluded at the title/abstract review phase. Should there be a disagreement in eligibility of the included articles, D.N. would resolve the discrepancy.

Studies evaluating the association between MPD, IPMN (including BD-IPMN, MD-IPMN, or mixed-type IPMN), and malignancy were included in this study. We specifically only included studies that reported MPD ranges that could be used to create two cut-offs: ≥5 mm and ≥10 mm. Studies also had to include the counts or rates of malignancy and non-malignancy for each MPD category. We also required that included articles show histological diagnosis of lesions, specifically whether they were non-malignant or malignant. In this study, histology classification and definition are in accordance with the 2015 Baltimore Consensus Meeting Guideline [23] or the current World Health Organization guidelines [24] for IPMN. In short, HGD or IC were defined as malignancy, whereas low-grade dysplasia or moderate-grade dysplasia were defined as non-malignancy. In the event of encountering two or more publications that had identical cohorts, only the publication with the most complete dataset was included in this study.

The primary outcomes of this study were the proportion of patients with HGD, IC, or malignancy.

### 2.2. Data Extraction

Study characteristics and data were independently extracted by two investigators (Y.H.A.W. and A.O.) and recorded on a standardized data extraction form. Any discrepancies were resolved by other reviewers (S.F., D.N., L.B., and K.L.C.). Data extracted and used for analysis are listed in Table 1. The final extracted data were reviewed by Y.H.A.W., A.O., S.F., L.B., and K.L.C.

### 2.3. Risk of Bias Analysis

Y.H.A.W., S.F., and A.O. assessed the quality of the included articles by conducting an evaluation using the Risk of Bias in Non-randomized Studies of Interventions (ROBINS-I) [41]. ROBINS-1 is designed to assess an article by evaluating 7 domains of risk of bias (Table S1). The assessment will grade the 7 domains of an article with low, moderate, serious, critical, or not assessable risk. In the end, the 7 domains were collectively analyzed for every article included in this study. Additionally, Funnel plots and Egger’s regression tests were used to assess publication bias (Figure S2).

### 2.4. Data Synthesis and Statistical Analysis

Tests of association: Random effects models were used to estimate adjusted diagnostic odds ratios (OR) and 95% confidence intervals for the pooled data. These were estimated using the meta package in RStudio (RStudio, Boston, Massachusetts), which provides functions for diagnostic meta-analysis [42]. A continuity correction was applied to all cells in a 2 × 2 table when necessary. We evaluated the association between cutoffs of ≥5 mm and ≥10 mm and classification of HGD, IC, and malignancy. There were three primary comparisons made for the two cutoffs: (1) classification of non-malignancy and malignancy; (2) classification of non-malignancy and HGD, and (3) classification of non-malignancy and IC. Therefore, we estimated six ORs using two cutoffs and three disease classifications. Forest Plots were used to compare individual-study ORs and the pooled OR. Forest plots include OR estimates from both the fixed effects model and the random effects model. Heterogeneity amongst the included publications was assessed using Cochrane’s Q test and I^2^. Based on the results of these tests, this paper utilized only the random effects estimates for interpretations.

Diagnostic tests: Pooled sensitivities and specificities and 95% confidence intervals for the same six comparisons were also estimated using the mada package (R Foundation for Statistical Computing, Vienna, Austria). Using a random effects model, the mada package implements a bivariate estimation of sensitivity and specificity described by Reitsma et al. [43]. This bivariate approach is necessary because the sensitivity and specificity of a test are interrelated; therefore, univariate approaches to estimation are inappropriate. From these pooled values for sensitivity and specificity, we then estimated AUC for each comparison. The summary receiver operating characteristic (SROC) curves were plotted to assess the spread of diagnostic measures for each comparison and cutoff.

We conducted an additional analysis to evaluate the impact of including the large study by Del Chiaro et al. (senior author of this study) [15]. This was done by repeating the analyses described above excluding that study.

## 3. Results

**Figure 1:**
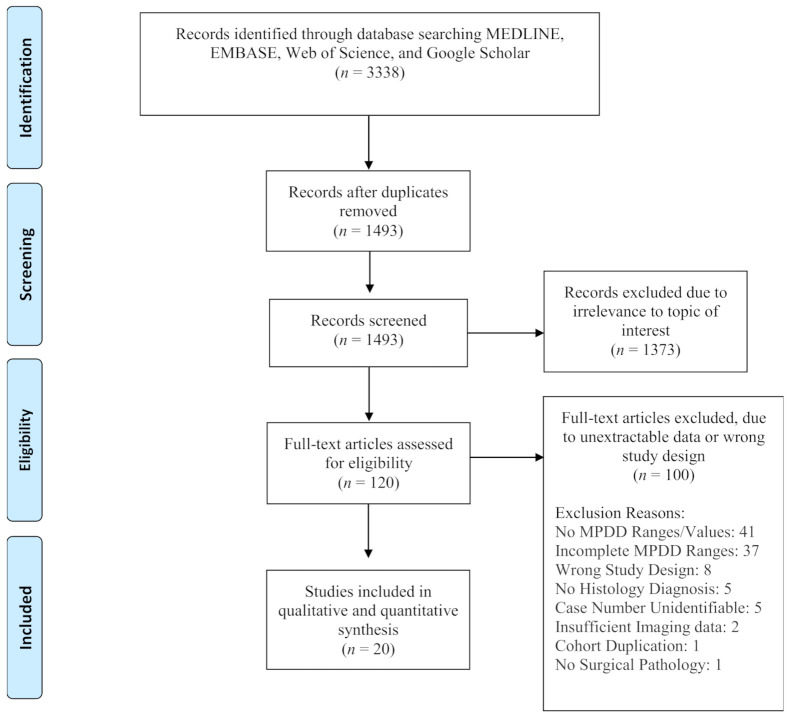
PRISMA flow chart showing the article selection process.

The search strategy identified 3338 citations. After removing duplicated articles, 1493 were eligible for title and abstract review. Initial title and abstract review performed by two independent reviewers (Y.H.A.W. and A.O.) yielded 120 articles eligible for full-article review. Ultimately, 20 manuscripts were included for qualitative and quantitative analysis. The authors excluded one hundred manuscripts for the following reasons: 41 articles did not have MPD dilatation values; 37 articles did not have all the MPD ranges that adhered to our criteria; 8 articles had incorrect study design and did not include information needed for quantitative meta-analysis; 5 articles had vague or no histology diagnosis; 5 articles did not provide sufficient information for case number retrieval; 2 articles only used ultrasound/endoscopic ultrasound for preoperative evaluation; 1 article lacked surgical pathology results; 1 article had duplication of cohort with another included article (Figure 1).

Characteristics of articles included for qualitative and quantitative analysis are listed in Table 1 and Table 2. From the 20 included articles [12,14,15,17,25,26,27,28,29,30,31,33,34,35,36,37,38,39,40,44,45], a total of 3982 resected IPMN cases (including BD-IPMN, MD-IPMN, or mixed-type IPMN) were collected. Of the 3982 resected cases, 1516 and 2466 were malignant and non-malignant cases, respectively. Of the 1516 malignant cases, 316, 680, and 520 cases had MPD range of <5 mm, 5–9.9 mm, and ≥10 mm, respectively. Similarly, of the 2466 non-malignant cases, 1404, 773, and 289 cases had MPD range of <5 mm, 5–9.9 mm, and ≥10 mm, respectively. Ten of the 20 studies included pathologically confirmed HGD and IC cases. Of the 818 malignant cases collected from the 10 articles, 421 and 397 cases had histology diagnosis of HGD and IC, respectively. Of the 421 HGD cases, 89, 187, and 145 cases had MPD range of <5 mm, 5–9 mm, and ≥10 mm, respectively. Similarly, of the 397 IC cases, 70, 182, 145 cases had MPD range of <5 mm, 5–9.9 mm, and ≥10 mm, respectively.

ROBINS-I indicated that the risk of bias of the included study was moderate in 18 [12,14,15,17,26,27,28,29,30,31,32,33,34,35,36,37,38,39,40] studies and serious in 2 [25,32]. The results of this analysis are provided in Table S1.

### 3.1. Tests of Association

**Figure 2:**
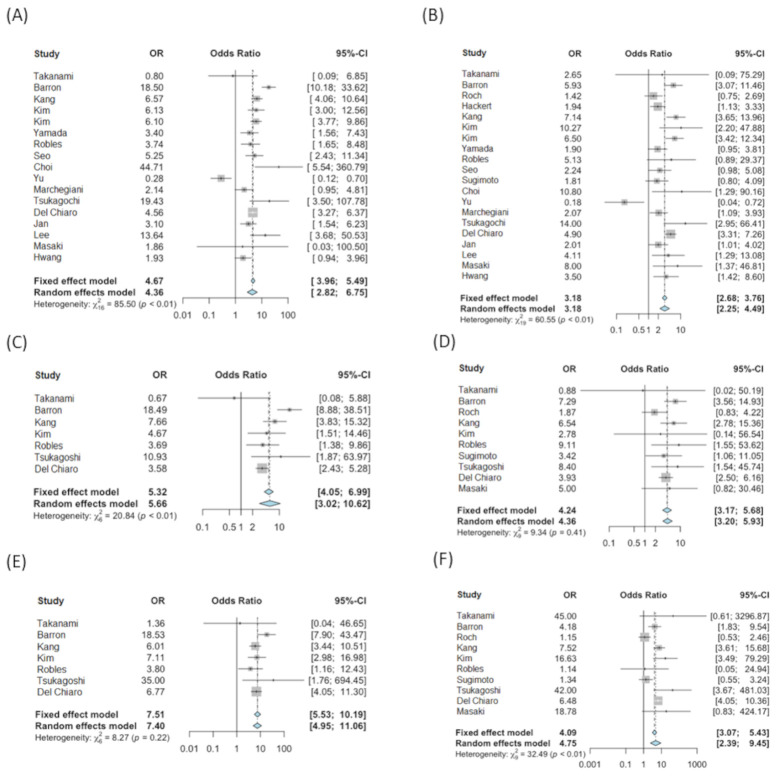
Meta-analysis forest plot presented in Diagnostic Odds Ratio (OR): (**A**) malignancy (M) and non-malignancy (NM), cutoff of 5 mm; (**B**) malignancy (M) and non-malignancy (NM), cutoff of 10 mm; (**C**) high-grade dysplasia (HGD) and non-malignancy (NM), cutoff of 5 mm; (**D**) high-grade dysplasia (HGD) and non-malignancy (NM), cutoff of 10 mm; (**E**) invasive carcinoma (IC) vs. non-malignancy (NM), cutoff of 5 mm; (**F**) invasive carcinoma (IC) vs. non-malignancy (NM), cutoff of 10 mm.

Both the ≥5 mm and ≥10 mm cutoffs in resected cases were significantly associated with higher risk of malignancy compared to <5 mm and <10 mm, respectively (≥5 mm: OR = 4.36 [95% CI: 2.82, 6.75, 6.75, I^2^ = 81.7%, Cochran’s Q *p* < 0.0001]; ≥10 mm: OR = 3.18 [95% CI: 2.25, 4.49, I^2^ = 68.2%, Cochran’s Q *p* < 0.0001]). The odds of HGD were over five times higher for patients with ≥5 mm MPD {5.66 (95% CI: 3.02, 10.62, I^2^ = 71.2%, Cochran’s Q *p =* 0.002)} compared to patients with MPD < 5 mm and over four times higher for patients with ≥10 mm MPD {4.36 (95% CI: 3.20, 5.93, I^2^ = 8.4%, Cochran’s Q *p =* 0.365)} compared to patients with <10 mm. The odds of IC were over seven times higher for patients with MPD ≥5 mm {7.40 (95% CI: 4.95, 11.06, I^2^ = 27.5%, Cochran’s Q *p =* 0.2189)} compared to <5 mm and 4.7 times higher for patients with ≥10 mm duct {4.75 (95% CI: 2.39, 9.45, I^2^ = 72.3%, Cochran’s Q *p =* 0.0002)} compared to <10 mm. Forest Plots are shown in Figure 2. Summary of the odds of HGD and IC for different MPD cutoffs are in Table S2.

### 3.2. Diagnostic Tests

**Figure 3:**
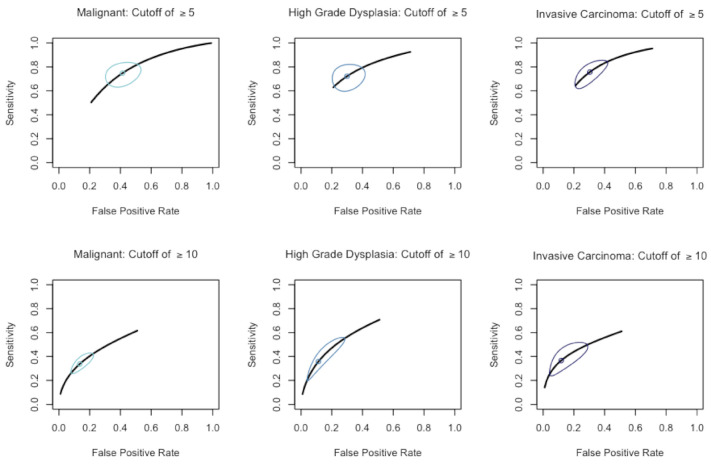
Summary receiver operating characteristic (SROC) curves for the bivariate diagnostic tests. Dot is point estimate that represents estimated pooled sensitivity/1-specificity for the included studies. Circle represents the confidence interval around the point estimate.

For all six comparisons, the tests for equality of sensitivities and specificities were all found to be significant (*p* < 0.001). This indicated heterogeneity between studies, and therefore we estimated pooled sensitivities and specificities using random effects models (Table 3). The six SROC curves from the random effects models are included in Figure 3.

Using a cutoff point of 5 mm, specificity was 58.6% and sensitivity was 74.8% for classification of malignancy. The AUC was 0.716. For the cutoff point of 10 mm, specificity was 86.4% and sensitivity were 33.8% for classification of malignancy. The AUC was 0.586. A cutoff of 5 mm had 70.1% specificity and 72.2% sensitivity for classification of HGD. The AUC was 0.769. A cutoff of 10 mm had 88.7% specificity and 35.7% sensitivity for classification of HGD. The AUC was 0.587. A cutoff point of 5 mm had 69.7% specificity and 75.6% sensitivity for classification of IC. The AUC was 0.786. A cutoff of 10 mm had 88.2% specificity and 36.6% sensitivity for classification of IC. The AUC was 0.587 (Figure 3).

### 3.3. Subset Analysis Excluding Del Chiaro et al.

The analysis excluding the large study by Del Chiaro et al. [15] yielded very similar results to the overall results presented in this study. The results of this analysis are provided in Table S2. 

## 4. Discussion

The correct clinical management of IPMNs is crucial for the prevention of pancreatic cancer [46]. An overtreatment of low-grade dysplasia lesions could result in unnecessary morbidity and mortality related to pancreatic surgery. Surgery is indicated for IPMNs in an attempt to remove IC and HGD. The latter is the optimal pre-invasive histology form and time-point for surgical intervention. Unfortunately, there is no available method that can effectively discriminate HGD from IC, except a few experimental approaches that are not yet implemented in clinical practice [15,47,48]. Recent studies reported that pancreatectomy-related mortality has decreased from 7.3% since 2000 [49], and the benchmark for postoperative mortality after pancreaticoduodenectomy published in 2019 was ≤1.6% [50], which is drastically lower than the 17–42% probability of five-year survival of resected IC [50,51,52,53,54]. Therefore, surgery performed on IC could be too late for extending the survival of patients, and it could be more beneficial to resect MD-IPMN while in HGD form.

According to the European evidence-based guideline for PCN management, IPMN MPD dilatation of 5–9.9 mm and ≥10 mm are relative and absolute indication for surgery, respectively [6]. Recent studies have demonstrated IPMN with MPD dilatation of ≥5 mm have a malignancy rate of 30–90% [12,15,28,33,55,56,57,58,59]. In this meta-analysis of 20 retrospective studies, malignancy was detected in nearly 46.8% of resected IPMN patients with 5–9.9 mm MPD and 63.5% of resected IPMN patients with ≥10 mm MPD. In total, 53.1% of resected IPMN patients with ≥5 mm MPD had malignancy. On the contrary, only 18.4% of resected cases with MPD < 5 mm were malignant IPMN. Pooled OR for malignancy calculated in this meta-analysis showed that MPD cutoff set at ≥5 mm was higher than ≥10 mm (OR = 4.4 vs. 3.2) in the resected cases. To improve the overall survival rate of malignant IPMN, HGD should be surgically removed before allowing it to progress to IC. Pooled OR from this meta-analysis showed that the odds of HGD were higher in ≥5 mm MPD than ≥10 mm MPD (OR = 5.7 vs. 4.4) in resected cases. Similarly, pooled OR for IC was also higher in ≥5 mm MPD than in ≥10 mm MPD (OR = 7.4 vs. 4.8). These data suggest that ductal dilatation of ≥5 mm should trigger the consideration for pancreatectomy.

The pooled sensitivities of ≥5 mm MPD in predicting IPMN HGD and IC were 72.2% and 75.6%, respectively. When using ≥5 mm MPD as cutoff, SROC AUC was 0.769 and 0.786 for HGD and IC, respectively, which were higher than those of ≥10 mm cutoff (AUC = 0.657 and 0. 587 for HGD and IC, respectively). The predictive role of ≥10 mm MPD for malignancy is unquestionable, but ≥5 mm MPD cutoff should also be considered as a highly sensitive factor for detecting HGD and/or IC. This lower cutoff could potentially identify malignancy in advance and improve survival of IPMN patients. However, Marchegiani et al. suggested that utilization of MPD dilatation as the sole indicator for pancreatectomy could encourage unnecessary surgical procedure and that most individuals with 5–9 mm MPD should be managed expectantly, performing conversion surgery when tumor progression is identified during close, regular follow-ups [17]. In the observation arm of the same study, 3 out of 46 (6.5%) IPMN patients with 5–9 mm MPD eventually underwent surgery [17]. However, Salvia et al. reported that the diagnostic accuracy for MD-IPMNs is only approximately 80% [19], implying some of those IPMN patients who underwent conservative treatment did not actually have IPMN [21]. If a PCN were to present with mural nodule(s) or cyst(s), fine-needle aspiration (FNA) with real-time endoscopic ultrasound (EUS) could be utilized to obtain histology proof and to make a diagnosis. It is important to note that EUS can accurately identify morphologic features of PCNs but is only 51% accurate in discerning the difference between mucinous and non-mucinous lesions [60]. The accuracy would increase up to 79% if CEA measurements were obtained from cystic fluid using EUS-FNA [60]. However, mural nodules and cysts ≥40 mm have only been reported in 1.2% to 21.3% and 23.2% to 27.5% of malignant IPMNs, respectively [13,15], and FNA would not have been a possible approach if ductal dilatation were the only cross-sectional imagining finding. Studies that include resected cases disproportionately favor inclusion of more serious cases that require surgery, but at least the diagnosis could be confirmed with surgical pathology. Including only pathologically confirmed IPMN cases for analysis, this study shows that many HGD and IC patients would be missed if the decision to operate were made at MPD ≥ 10 mm associated with low sensitivity.

The results in this study underline the need for a surgical evaluation for MPD ≥5 mm and are not conclusive evidence that suggest the risk for developing cancer is higher in IPMN with 5–9.9 mm MPD. On the other hand, it could also be possible that IPMN with MPD dilatation over 10 mm are slow progressive diseases that would turn invasive when MPD reaches larger dilatation. Using ≥5 mm MPD to select surgical candidates might also identify low-grade dysplasia. Considering that the highest incidence of IC occurs with MD-IPMN, we could assume that the surgical treatment of MD-IPMN with low-grade dysplasia is perhaps not an overtreatment when compared to surgical treatment of BD-IPMN with low-grade dysplasia [21]. Nevertheless, it is important to know that the currently available information for IPMN management is not perfect, and it is important to diagnose IPMN with existing sensitive diagnostic tools to increase the overall survival rates of the patients, as the mean frequency of malignancy in MPD dilatation of ≥5 mm was 61.6% and that of IC IPMN was 43.1% [11].

Recognizing the potential for the Del Chiaro et al. study [15] of heavily influencing the results presented in this study given its overall size, we completed a full analysis of the data excluding this study. The results and conclusions were unchanged.

Our study has limitations related to the use of retrospective studies that lack conservatively managed cohorts as a control group. Cases could not be stratified based on the imaging modalities used. The most accurate cutoff line for MPD dilatation could not be determined in this study since data for MPD dilatation are presented in ranges. In addition, other features associated with IPMN malignancy, such as mural nodule, cytology, elevated CA 19–9, or cyst diameter, could not be included in this analysis [11]. In addition, this study includes only resected IPMNs. Therefore, the results of this study are not representative of the unresected IPMN population. However, this study was performed based on recently published study that showed MPD dilatation is currently the best predictor of HGD or IC in IPMN [15]. Although estimates of heterogeneity above 50% were observed for some of our analyses, we found no reason for publication bias based on visual inspection of the funnel plot and the results of the Egger’s test (Egger Regression Model). Additionally, and as stated in the methodology, all of our statistical models were estimated using random model effects, which have previously been used successfully to account for this limitation [42]. Even with some limitations related to the retrospective nature of this study, this analysis contains the largest volume of IPMN cases analyzed and our methodological approach properly accounted for study-level variation. 

## 5. Conclusions

MPD dilatation is an important predictive factor of IPMN malignancy and 5 mm is a highly sensitive cutoff that detects high-risk pre-cancerous or cancerous lesions in resected cases. It is important to note that this study cannot draw a conclusion for non-surgical cases. However, the need for pancreatectomy should be thoroughly evaluated in patients with ductal dilatation of ≥5 mm. The result of this study implies that MPD dilatation over 5 mm should trigger the referral of a patient to a high-volume center for further consultation. The decision to perform resection should only be considered after careful evaluation of multiple aspects related to the general conditions and the expectancy of life of a patient. In conjunction with new biomarkers or diagnostic modalities such as pancreatoscopy [61,62], MPD dilatation detection could improve surgical patient selection and reduce overall IPMN malignancy mortality.

## Figures and Tables

**Table 1:** Characteristics of included articles.

Author	Year	Country	Design	<5 mm (*n*)		5–9 mm (*n*)		≥10 mm (*n*)	
				M	NM	M	NM	M	NM
Takanami et al. [25]	2011	Japan	Retrospective	3	2	5	5	1	0
Barron et al. [26]	2014	U.S.A.	Retrospective	17	149	74	40	40	14
Roch et al. [27]	2014	U.S.A.	Retrospective	-	-	50	64	30	27
Hackert et al. [12]	2015	Germany	Retrospective	-	-	93	64	76	27
Kang et al. [28]	2015	S. Korea	Retrospective	44	206	39	38	34	14
Kim et al. [29]	2015	S. Korea	Retrospective	15	212	19	50	4	3
Kim et al. [30]	2015	S. Korea	Retrospective	43	195	38	39	36	16
Yamada et al. [31]	2015	Japan	Retrospective	10	42	29	39	22	24
Robles et al. [32]	2016	France	Retrospective	13	57	19	25	4	2
Seo et al. [33]	2016	S. Korea	Retrospective	11	62	27	29	14	15
Sugimoto et al. [14]	2016	U.S.A.	Retrospective	-	-	22	19	42	20
Choi et al. [34]	2017	S. Korea	Retrospective	1	20	29	16	9	1
Yu et al. [35]	2017	Japan	Retrospective	39	13	14	12	3	8
Marchegiani et al. [17]	2018	Italy	Retrospective	8	43	43	126	20	32
Tsukagoshi et al. [36]	2018	Japan	Retrospective	2	17	4	4	12	3
Del Chiaro et al. [15]	2019	U.S.A./Sweden	Retrospective	65	240	134	152	107	43
Jan et al. [37]	2019	Taiwan	Retrospective	17	65	11	11	23	31
Lee et al. [38]	2019	S. Korea	Retrospective	3	36	16	16	9	6
Masaki et al. [39]	2019	Japan	Retrospective	0	0	3	6	16	4
Hwang et al. [40]	2020	S. Korea	Retrospective	25	45	11	18	18	9
Total (% *)				316 (18.4%)	1404 (81.6%)	680 (46.8%)	773 (53.2%)	520 (63.5%)	289 (36.5%)

* Percentage of cases within MPD Dilatation category; Abbreviations: M = Malignancy; NM = Non-Malignancy.

**Table 2:** Characteristics of included article with postoperative histology diagnosis of HGD and IC.

Author	Year	Design	<5 mm			5–9 mm			≥10 mm		
			HGD	IC	NM	HGD	IC	NM	HGD	IC	NM
Takanami et al. [25]	2011	Retrospective	3	0	2	5	0	5	0	1	0
Barron et al. [26]	2014	Retrospective	10	7	149	40	34	40	27	13	14
Roch et al. [27]	2014	Retrospective	-	-	-	19	31	64	15	15	27
Kang et al. [28]	2015	Retrospective	15	29	206	17	22	38	12	22	14
Kim et al. [29]	2015	Retrospective	6	9	212	7	12	50	0	4	3
Robles et al. [32]	2016	Retrospective	8	5	57	10	9	25	4	0	2
Sugimoto et al. [14]	2016	Retrospective	-	-	-	5	17	19	18	24	20
Tsukagoshi et al. [36]	2018	Retrospective	2	0	17	3	1	4	6	6	3
Del Chiaro et al. [15]	2019	Retrospective	45	20	240	78	56	152	53	54	43
Masaki et al. [39]	2019	Retrospective	0	0	0	3	0	6	10	6	4
Total (% *)			89 (8.5%)	70 (6.7%)	883 (84.7%)	187 (24.2%)	182 (23.6%)	403 (52.2%)	145 (34.5%)	145 (34.5%)	130 (31.0%)

* Percentage of cases within MPD Dilatation category; Abbreviations: HGD, High Grade Dysplasia; IC, Invasive Carcinoma; NM, Non-Malignancy.

**Table 3:** Pooled sensitivity/specificity and area under the curve (AUC).

Comparisons	Dilation	Sensitivity	95% CI	Specificity	95% CI	AUC	Studies Included
**Malignancy to NM**	≥5 mm	74.8%	(64.6–82.2%)	58.6%	(49.0–67.6%)	0.716	17
	≥10 mm	33.8%	(27.2–41.0%)	86.4%	(79.6–91.2%)	0.586	20
**High-Grade Dysplasia to NM**	≥5 mm	72.2%	(62.2–80.3%)	70.1%	(60.7–78.0%)	0.769	7
	≥10 mm	35.7%	(22.3–51.9%)	88.7%	(75.8–95.1%)	0.657	10
**Invasive Carcinoma to NM**	≥5 mm	75.6%	(64.8–83.9%)	69.7%	(60.4–77.6%)	0.786	7
	≥10 mm	36.6%	(26.0–48.7%)	88.2%	(75.3–94.9%)	0.587	10

Abbreviations: NM, Non-Malignancy.

## Supplementary Materials

The following are available online at https://www.mdpi.com/article/10.3390/cancers13092031/s1. Figure S1: Detailed Search Strategy for Existing Online Literature, Figure S2: Funnel plot with Diagnostic Odds Ratio (DOR) and Egger’s Test for publication bias assessment, Table S1: ROBINS-I Risk of Bias Assessment of included Articles, Table S2: Odds ratio, Sensitive, and Specificity for different comparisons and cutoffs.
