# Supplementary material for: Ductal Dilatation of ≥5 mm in Intraductal Papillary Mucinous Neoplasm Should Trigger the Consideration for Pancreatectomy: A Meta-Analysis and Systematic Review of Resected Cases
Source: Cancers (Basel). 2021 Apr 22;13(9):2031. doi: 10.3390/cancers13092031 (PMC8122854; doi:10.3390/cancers13092031)
Supplement: Supplementary file 1 [file cancers-13-02031-s001.zip › cancers-1148973-supplementary.pdf]

cancers

MDPI

Supplementary

Ductal Dilatation of ≥5 mm in Intraductal Papillary Mucinous Neoplasm Should Trigger the Consideration for Pancreatectomy: A Meta-Analysis and Systematic Review of Resected Cases

Y.H. Andrew Wu et. al.

The following search strategy was used:
Database(s): Ovid MEDLINE(R) ALL 1946 to February 27, 2020
Search Strategy:

#	Searches	Results
1	(intraductal papillary mucinous or intraductal mucinous papillary or ipmn* or ipmt*).tw,kf. or exp Pancreatic Intraductal Neoplasms/	3326
2	(dilat* or size* or diameter* or cut off* or cutoff* or mm or millimeter* or milli meter* or ((radiographic* or CT or comput* tomograph* or comput* axial tomograph* or MRI or magnetic resonance imag* or magnetic resonance tomograph* or MRCP or magnetic resonance cholangiopancreatograph* or imag*) adj2 (anlys* or determination or assessment* or measure*))).tw,kf.	2015842
3	1 and 2	1003
4	remove duplicates from 3	1002

Database(s): Embase Suite: Embase (1974 to February 28, 2020); Medline (1966 to February 28, 2020); Embase Classic (1947 to 1973).
Search Strategy:

#	Searches	Results
1	'intraductal papillary mucinous':ti,ab OR 'intraductal mucinous papillary':ti,ab OR ipmn*:ti,ab OR ipmt*:ti,ab OR 'intraductal papillary mucinous tumor'/exp	7656
2	dilat*:ti,ab OR size*:ti,ab OR diameter*:ti,ab OR 'cut off*':ti,ab OR cutoff*:ti,ab OR mm:ti,ab OR millimeter*:ti,ab OR 'milli meter*':ti,ab OR (((radiographic* OR ct OR 'comput* tomograph*' OR 'comput* axial tomograph*' OR mri OR 'magnetic resonance imag*' OR 'magnetic resonance tomograph*' OR mrcp OR 'magnetic resonance cholangiopancreatograph*' OR imag*) NEAR/2 (anlys* OR determination OR assessment* OR measure*)):ti,ab)	2681414
3	#1 AND #2	2591
4	#1 AND #2 AND ([conference abstract]/lim OR [conference paper]/lim OR [conference review]/lim)	1418
5	#3 NOT #4	1173

Database(s): Web Science (Science Citation Index Expanded, 1900 to February 28, 2020; Social Sciences Citation Index,1900 to February 28, 2020).
Search Strategy:

#	Searches	Results
1	TS=("intraductal papillary mucinous" or "intraductal mucinous papillary" or ipmn* or ipmt*)	3,860
2	TS=(dilat* or size* or diameter* or "cut off*" or cutoff* or mm or millimeter* or "milli meter*" or (radiographic* or CT or "comput* tomograph*" or "comput* axial tomograph*" or MRI or "magnetic resonance imag*" or "magnetic resonance tomograph*" or MRCP or "magnetic resonance cholangiopancreatograph or imag*) near/2 (anlys* or determination or assessment* or measure*))	3,658,238
3	#2 AND #1	3 863

Database(s): Google Scholar.

Search Strategy:.

#	Searches	Results
1	intraductal papillary mucinous neoplasm duct diameter∣size image analysis∣measure	1st 300

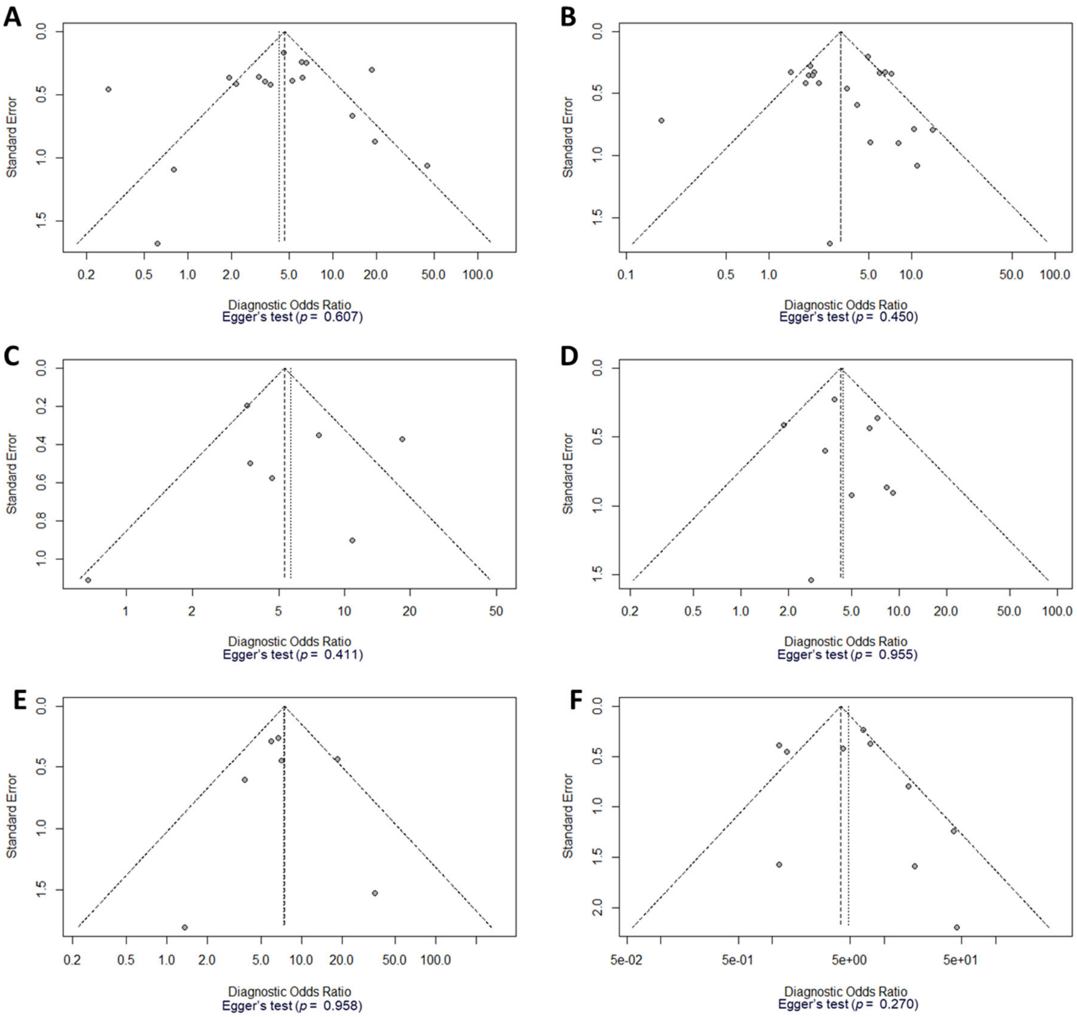

Figure S2. Funnel plot with Diagnostic Odds Ratio (DOR) and Egger's Test for publication bias assessment: A: Malignancy (M) and non-malignancy (NM), cutoff of 5 mm; B: Malignancy (M) and non-malignancy (NM), cutoff of 10 mm; C: High-grade dysplasia (HGD) and non-malignancy (NM), cutoff of 5 mm; D: High-grade dysplasia (HGD) and non-malignancy (NM), cutoff of 10 mm; E: Invasive carcinoma (IC) vs. non-malignancy (NM), cutoff of 5 mm; F: Invasive carcinoma (IC) vs. non-malignancy (NM), cutoff of 10 mm.

Table S1 ROBINS-I Risk of Bias Assessment of included Articles									
**Author**	**Year**	**Confounding**	**Participants Selection**	**Intervention Classification**	**Intended Intervention Deviation**	**Missing Data**	**Outcome Measurement**	**Selective Reporting**	**Overall**
K. Takanami et al.	2011	Serious	Serious	Moderate	Low	Serious	Low	Serious	Serious
A. Roch et al.	2014	Serious	Serious	Moderate	Low	Moderate	Low	NR	Moderate
M. Barron et al.	2014	Serious	Serious	Moderate	Low	NR	Low	NR	Moderate
T. Hackert et al.	2015	Serious	Serious	Moderate	Low	Moderate	Low	NR	Moderate
Y. Kim et al.	2015	Serious	Serious	Moderate	Low	Moderate	Low	NR	Moderate
S. Yamada et al.	2015	Serious	Serious	Moderate	Low	Moderate	Low	Moderate	Moderate
J. Kim et al.	2015	Serious	Serious	Moderate	Low	NR	Low	NR	Moderate
M. Kang et al.	2015	Serious	Serious	Moderate	Low	NR	Low	NR	Moderate
N. Seo et al.	2016	Serious	Serious	Moderate	Low	Moderate	Low	NR	Moderate
M. Sugimoto et al.	2016	Serious	Serious	Moderate	Low	Moderate	Low	NR	Moderate
E. Robles et al.	2016	Serious	Serious	Moderate	Moderate	Serious	Low	NR	Serious
S. Yu et al.	2017	Serious	Serious	Moderate	Low	NR	Low	NR	Moderate
S. Choi et al.	2017	Serious	Serious	Moderate	Low	Moderate	Moderate	NR	Moderate
M. Tsukagoshi et al.	2018	Serious	Serious	Moderate	Low	NR	Low	NR	Moderate
G. Marchegiani et al.	2018	Serious	Serious	Moderate	Low	NR	Low	Moderate	Moderate
Y. Masaki et al.	2019	Serious	Serious	Moderate	Low	Moderate	Low	NR	Moderate
I. Jan et al.	2019	Serious	Serious	Moderate	Low	NR	Low	NR	Moderate
M. Del Chiaro et al.	2019	Serious	Serious	Moderate	Low	Moderate	Moderate	NR	Moderate
J. Lee et al.	2019	Serious	Serious	Moderate	Low	Moderate	Low	NR	Moderate
J. Hwang et al.	2020	Serious	Serious	Moderate	Moderate	NR	Low	NR	Moderate

Abbreviations: NR, Not Reported.

Table S2 Odds ratio, Sensitive, and Specificity for different comparisons and cutoffs									
	**Malignancy to NM**			**High Grade Dysplasia to NM**			**Invasive Carcinoma to NM**		
**Cutoffs**	**OR**	**Sensitivity**	**Specificity**	**OR**	**Sensitivity**	**Specificity**	**OR**	**Sensitivity**	**Specificity**
**≥ 5 mm**	4.36	74.8%	58.6%	5.66	72.2%	70.1%	7.4	75.6%	69.7%
**≥ 10 mm**	3.18	33.8%	86.4%	4.36	35.7%	88.7%	4.75	36.6%	88.2%
**≥ 5 mm***	4.36*	74.6%*	58.7%*	6.39*	71.8%*	75.0%*	7.72*	74.4%*	74.6%*
**≥ 10 mm***	3.08*	33.3%*	86.1%*	4.65*	38.0%*	88.9%*	4.68*	34.5%*	87.8%*

Abbreviations: NM, Non-Malignancy
* Indicates the estimates with the M. Del Chiaro et al. study removed.
